# Morphological Ontogeny, Ecology, and Biogeography of *Fuscozetes fuscipes* (Acari, Oribatida, Ceratozetidae)

**DOI:** 10.3390/ani14040538

**Published:** 2024-02-06

**Authors:** Stanisław Seniczak, Anna Seniczak, Bjarte H. Jordal

**Affiliations:** 1 Department of Evolutionary Biology, Faculty of Biological Sciences, Kazimierz Wielki University, 85-093 Bydgoszcz, Poland; stseni@ukw.edu.pl; 2 Faculty of Applied Ecology, Agricultural Sciences and Biotechnology, Inland Norway University of Applied Sciences, 2318 Elverum, Norway; 3 University Museum of Bergen, University of Bergen, 5007 Bergen, Norway; bjarte.jordal@uib.no

**Keywords:** oribatid mites, Sphaerozetinae, juveniles, leg setation, stage structure, DNA barcoding

## Abstract

**Simple Summary:**

The systematic status of *Fuscozetes* is not clear in the literature. Therefore, the morphological ontogeny of *F*. *fuscipes*, the type species of this genus, was investigated and compared with its congeners in this study, and a new diagnosis of *Fuscozetes* is given. The juveniles of *F*. *fuscipes* are light brown, with a brown prodorsum, sclerites, epimeres, and legs. In all juveniles, a humeral organ and a humeral macrosclerite are present. The gastronotum of the larva has 12 pairs of setae (*h*_3_ is present), while the nymphs have 15 pairs. In the larva, the gastronotal shield is weakly developed, most of the gastronotal setae are short and inserted on the microsclerites, and several other macrosclerites and many microsclerites are present on the hysterosoma. In the nymphs, the gastronotal shield is well developed, with 10 pairs of setae (*d*-, *l*-, and *h*-series, and *p*_1_), and setae *p*_2_ and *p*_3_ are located on a large posteroventral macrosclerite. In all the instars, femora I and II are oval in cross-section, without a large ventral carina. Mitochondrial COI sequence data revealed a deep split between the Nearctic and Palearctic populations of *F*. *fuscipes*, and a less, but significant, divergence within each continent. These strong geographical barriers were contrasted with multiple cases of shared haplotypes over long distances in the Palearctic, indicating high migration rates in modern times.

**Abstract:**

The systematic status of *Fuscozetes* Sellnick, 1928, is not clear in the literature. Therefore, the morphological ontogeny of *F*. *fuscipes* (C.L. Koch, 1844), the type species of this genus, was investigated and compared with its congeners in this study, and a new diagnosis of *Fuscozetes* is given. The juveniles of *F*. *fuscipes* are light brown, with a brown prodorsum, sclerites, epimeres, and legs. In all juveniles, a humeral organ and a humeral macrosclerite are present. The gastronotum of the larva has 12 pairs of setae (*h*_3_ is present), whereas the nymphs have 15 pairs. In the larva, the gastronotal shield is weakly developed, and most gastronotal setae are short except for a slightly longer *h*_2_. Most of the gastronotal setae are inserted on the microsclerites except for *h*_3_, and several other macrosclerites and many microsclerites are present on the hysterosoma. In the nymphs, the gastronotal shield is well developed, with 10 pairs of setae (*d*-, *l*-, and *h*-series, and *p*_1_), and setae *p*_2_ and *p*_3_ are located on a large posteroventral macrosclerite. In all the instars, femora I and II are oval in cross-section, without a large ventral carina. Mitochondrial COI sequence data revealed a deep split between the Nearctic and Palearctic populations of *F*. *fuscipes*, and a less, but significant, divergence within each continent. These strong geographical barriers were contrasted with multiple cases of shared haplotypes over long distances in the Palearctic, indicating high migration rates in modern times.

## 1. Introduction

*Fuscozetes* Sellnick, 1928, with the type species *F. fuscipes* (C.L Koch, 1844), is an average genus in terms of its number of species. It includes 15 species according to Subías [1], and two of them are considered *species inquirendae*. *Fuscozetes* belongs to the subfamily Spherozetinae sensu Shaldybina [2], which also contains the genera *Edwardzetes* Berlese, 1913; *Ghilarovizetes* Shaldybina, 1969; *Melanozetes* Hull, 1916; and *Sphaerozetes* Berlese, 1885.

The systematics of *Fuscozetes* are not clear in the literature, and its diagnosis has varied over time. Sellnick [3] paid particular attention to the skeletal characters of adults, whereas Shaldybina [2] added the number of notogastral setae, which can provide information on the phylogeny of moss mites [4,5,6]. Based on the morphology of the juvenile stages and adults of the *Fuscozetes* species, Shaldybina [2,7,8] limited the number of setae on the notogaster of adults to 10 or 11 pairs. Behan-Pelletier [9,10] expanded the diagnosis of *Fuscozetes* to 10–14 pairs, including *c*_3_, but this diagnosis was problematic because it also included *Melanozetes* Hull, 1916, which has 14 pairs of notogastral setae. Based on the morphology of the juvenile stages and adults of the *Fuscozetes* species, Seniczak et al. [11] restricted the diagnosis of *Fuscozetes* to 10–13 pairs of notogastral setae, including seta *c*_2_, and some or all the setae of the *d*-series. Next, Seniczak et al. [12] and Seniczak and Seniczak [13,14] added the length and position of solenidion ω_2_ on tarsus I, which clearly separated *Fuscozetes* from *Melanozetes*, both in the nymphs and the adults. In *Fuscozetes*, solenidion ω_2_ is shorter than ω_1_ and is placed posterolaterally to ω_1_, whereas in *Melanozetes*, solenidion ω_2_ is as long as or longer than ω_1_ and is placed anteriorly to ω_1_.

Shaldybina [2] included *Fuscozetes* in the subfamily Sphearozetinae, which clearly differs from Ceratozetinae and Trichoribatinae in both the adult and juvenile stages. Weigmann [15] omitted this proposal and included *Fuscozetes* and related genera in Ceratozetidae, indicating that the systematics of this family need further investigation.

The juvenile instars of the *Fuscozetes* species are relatively well studied. According to the catalogue by Norton and Ermilov [16], Seniczak et al. [12], and Seniczak and Seniczak [13,14], the morphological ontogeny of seven species of *Fuscozetes* is known: *F*. *coulsoni* A. and S. Seniczak, 2020; *F*. *fuscipes*; *F*. *kamchatkicus* Seniczak et al., 2016; *F*. *pseudosetosus* Shaldybina, 1969; *F*. *setiger* (Trägårdh, 1910); *F*. *setosus* (C.L. Koch, 1839); and *F*. *tatricus* Seniczak, 1993. The morphological ontogeny of *F*. *fuscipes* has already been investigated by Seniczak [17], but this study was general and omitted the lateral aspect of the larvae, tritonymphs, and adults, as well as the leg setation, which is important for the morphological comparison of species within the genus. Here, we investigated specimens of *F*. *fuscipes* from Norway, which differed slightly from those studied by Seniczak [17] from Poland, illustrating some regional variability in this species. We also illustrated the morphological structures of *F*. *fuscipes* with SEM figures to clarify the miniscule characters of this species. In addition, we compared the molecular data (COI) of *F*. *fuscipes* from different locations, based on our own and public data.

## 2. Materials and Methods

### 2.1. Morphological and Biological Studies

The specimens of *F*. *fuscipes* used in this study were collected on 15 June 2018 by A. Seniczak from patches of *Sphagnum* mosses on the shore of lake Skomakerdiket (Bergen, Vestland, Norway, 60°23′39.7″ N, 5°21′04.7″ E, 320 m a. s. l.). The samples were extracted in Berlese funnels in the laboratory of the Department of Natural History, University Museum of Bergen (Norway), over ten days. The juveniles of *F*. *fuscipes* were identified using the specific characters given by Seniczak [17]. In 30 adults selected at random, the sex ratio and the number of gravid females were determined, as well as the body length and width. We also measured the morphological characters of all the instars of *F*. *fuscipes*, namely the total body length (in the lateral aspect, from the tip of the rostrum to the posterior edge of the notogaster), the width (in the dorsal aspect, at the widest part of the notogaster), and the length of the anal and genital openings and the setae, perpendicularly to their length. All measurements are given in µm. All light microscopy was performed using a Nicon Eclipse Ni.

We illustrated the dorsal and lateral aspects of the larvae, tritonymphs, and adults; some leg segments of these stages; the ventral regions of all instars; and the palps and chelicerae of the adults. The illustrations were prepared from individuals mounted temporarily on slides in lactic acid. In the text and figures, the following abbreviations are used: rostral (*ro*), lamellar (*le*), interlamellar (*in*) and exobothridial (*ex*) setae, lamella (*La*), translamella (*Tr*), bothridium (*bo*), bothridial seta (*bs*), notogastral or gastronotal setae (*c*-, *d*-, *l*-, *h*-, and *p*-series), porose areas (*Aa*, *A1*, *A2*, and *A3*), opisthonotal gland opening (*gla*), pteromorph (*Ptm*), cupules or lyrifissures (*ia*, *im*, *ip*, *ih*, *ips*, and *iad*), tutorium (*Tut*), pedotectum (*Pd*), circumpedal carina (*cp*), custodium (*cus*), discidium (*Dis*), humeral sclerite (*hs*), humeral organ (*oh*), subcapitular setae (*a*, *m*, and *h*), cheliceral setae (*cha* and *chb*), Trägårdh organ (*Tg*), palp setae (*sup*, *inf*, *l*, *d*, *cm*, *acm*, *it*, *vt*, *ul*, and *su*), solenidion ω, adanal and anal setae (*ad*- and *an*-series), epimeral setae (*1a*–*c*, *2a*, *3a*–*c*, and *4a*–*c*), genital (*g*) and aggenital (*ag*) setae, leg solenidia (σ, φ, and ω), famulus (ε), and setae (*bv*, *d*, *l*, *ft*, *tc*, *it*, *p*, *u*, *a*, *s*, *pv*, *pl*, and *v*). The terminology used follows that of Grandjean [4,5,18,19,20,21], Behan-Pelletier [9,10], and Norton and Behan-Pelletier [22]. The species nomenclature follows Subías [1] and Norton and Ermilov [16].

For scanning electron microscopy (SEM), the mites were air-dried, coated with Au/Pd in a Polaron SC502 sputter-coater, and placed on Al stubs with double-sided sticky carbon tape. The observations and micrographs were made with a Quanta™ 450 FEG scanning electron microscope.

### 2.2. DNA Barcoding

The specimens of *F*. *fuscipes* used for molecular studies were collected in Southern, Central, and Northern Norway. We also used public sequences from the BOLD database, which originated from Canada, Finland, and Germany. The outgroup sequences represented two other *Fuscozetes* species, *F*. *setosus* and *F. setiger*, and representatives of all the other genera of the subfamily Spherozetinae (*Edwardzetes* Berlese, 1913; *Ghilarovizetes* Shaldybina, 1969; *Melanozetes* Hull, 1916; and *Sphaerozetes* Berlese, 1885) and representatives of two other related subfamilies, Trichoribatinae (*Diapterobates* Grandjean, 1936; *Neogymnobates* Ewing, 1917; *Svalbardia* Thor, 1930; and *Trichoribates* Berlese, 1910) and Ceratozetinae (*Ceratozetes* Berlese, 1908).

The specimens of *F*. *fuscipes* were DNA-barcoded at the Canadian Centre for DNA Barcoding (CCDB) in Guelph, Canada. Before sending the samples to the CCDB, each specimen was photographed, and these photos are vouchers available at the Barcode of Life Data System (BOLD, http://boldsystems.org, accessed on 20 November 2023). The specimens were placed in a well containing 50 mL of 90% ethanol in a 96-well microplate and submitted to the CCDB. The mites were sequenced for the barcode region of the COI gene according to standard protocols at the CCDB (www.ccdb.ca, accessed on 20 October 2020), using either the LepF1/LepR1 [23] or the LCO1490/m HCO2198 [24] primer pairs. The DNA extracts were placed in archival storage at −80 °C, mainly at the CCDB, and some (sequencing code starting with UMNFO) at the University Museum of Bergen (ZMUB). The COI sequence chromatograms were checked for double peaks and potential NUMTs, and were blasted in GenBank to detect and exclude possible contaminants. The sequences are available in GenBank (Table 1).

The sequences were aligned by eye in MEGA11: Molecular Evolutionary Genetics Analysis, version 11 [29]. A Bayesian inference (BI) analysis was conducted in MrBayes 3.2 using a GTR + G + I model of nucleotide substitutions [30]. Posterior probabilities were generated from 10 million generations of sampling from two independent runs using one cold (temp = 0.3) and three heated chains, excluding the first 25% of generations as burn-in. The chain convergence was assessed using a standard deviation of the split frequencies approaching 0.01 and a potential scale reduction factor (PSRF) of 1.0. The consensus tree topology was visualized in FigTree 1.4.2 (available at http://tree.bio.ed.ac.uk/software/figtree, accessed on 20 November 2023) and edited in Adobe Illustrator.

## 3. Results

### 3.1. Diagnosis of Fuscozetes Sellnick, 1928

Based on the morphological characters given by Seniczak et al. [11,12] and Seniczak and Seniczak [13,14] and *F. fuscipes* studied herein, the diagnosis of *Fuscozetes* is as follows: the adults are of a medium size (423–897), brown to dark brown, and with characters of Ceratozetidae [2]. A translamella is present or absent, the lamellar cusp is rounded or with teeth, and the bothridial seta is clavate or fusiform. The notogastral setae are short to long (10–13 pairs, including *c*_2_ and all or some setae of the *d*-series), and the porose areas (4 pairs) are of a similar size or with a larger *Aa*. Femora I and II are oval in cross-section, and solenidion ω_2_ on tarsus I is shorter than ω_1_ and is located posterolaterally to solenidion ω_1_.

The juveniles are light brown with a brown prodorsum, sclerites, epimeres, and legs. The bothridial seta is clavate or fusiform and the gastronotal setae are short to long. In the larva, a humeral organ and a humeral macrosclerite are present or absent. The gastronotal seta *c*_1_ is inserted on the humeral macrosclerite or microsclerite, or on the unsclerotized integument, while setae *c*_2_ and *c*_3_ are inserted on the microsclerites or the unsclerotized integument. The gastronotal shield is uniform, divided in two parts, structured as a pygidium, or absent (in which case most gastronotal setae are located on microsclerites); other sclerites and microsclerites are present or absent. In the nymphs, the humeral macrosclerite is present, whereas the humeral organ is present or absent. The gastronotal shield has 10 pairs of setae (*d*-, *l*-, and *h*-series, and *p*_1_), where setae *p*_2_ and *p*_3_ are placed on the macrosclerite or the unsclerotized integument. In all the juveniles, the femora are oval in cross-section, without a large ventral carina. Solenidion ω_2_ on tarsus I is shorter than solenidion ω_1_, and is located posterolaterally to ω_1_.

### 3.2. Morphological Ontogeny of Fuscozetes fuscipes (C.L. Koch, 1844) (Figure 1, Figure 2, Figure 3, Figure 4, Figure 5, Figure 6, Figure 7, Figure 8, Figure 9, Figure 10, Figure 11, Figure 12, Figure 13, Figure 14, Figure 15, Figure 16, Figure 17, Figure 18, Figure 19, Figure 20 and Figure 21)

*Oribata fuscipes* C.L. Koch, 1844: Michael [31].

*Fuscozetes fuscipes*: Sellnick [3], Willmann [32], Shaldybina [2], Mehl [33], Karppinen and Krivolutsky [34], Golosova et al. [35], Karppinen et al. [36,37], Marshall et al. [38], Olszanowski et al. [39], Niemi et al. [40], Ryabinin and Pankov [41], Subías [1], Weigmann [15], Miko [42], Murvanidze and Mumladze [43], Schatz [44], and Murvanidze et al. [45].

#### 3.2.1. Diagnosis

The adults are brown, of a medium size (629–897), and with the characters of *Fuscozetes* given above. The translamella is present, the lamellar cusp is long with teeth, and the bothridial seta is fusiform. The notogastral setae are long (10 pairs, including *c*_2_), and porose area *Aa* is rounded and slightly larger than the other porose areas.

In the juveniles, the bothridial seta is fusiform and a humeral organ and humeral macrosclerite are present. In the larva, the gastronotal shield is absent; most of the gastronotal setae are short and inserted on the microsclerites; and other sclerites and microsclerites are also present. In the nymphs, the gastronotal shield is present, the setae of the *c*-series are located on the microsclerites, and setae *p*_2_ and *p*_3_ are placed on a large, posteroventral sclerite.

#### 3.2.2. Morphology of Adults

**Figure 1:**
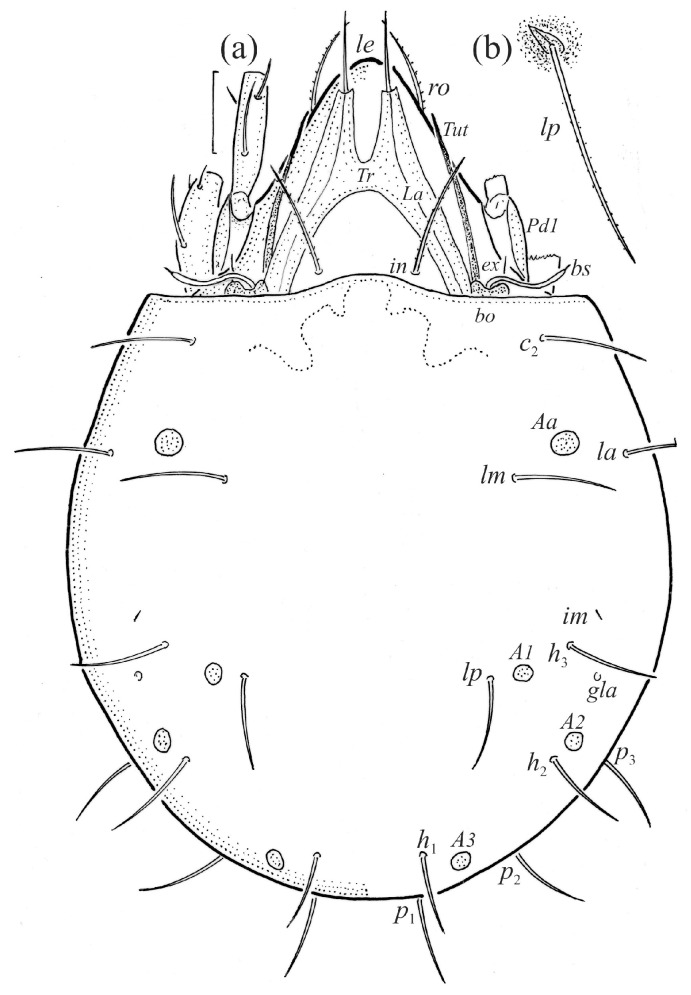
Adult female *Fuscozetes fuscipes*. (**a**) Dorsal aspect, with legs partially drawn; scale bar: 50 μm. (**b**) Seta *lp* (enlarged).

**Figure 2:**
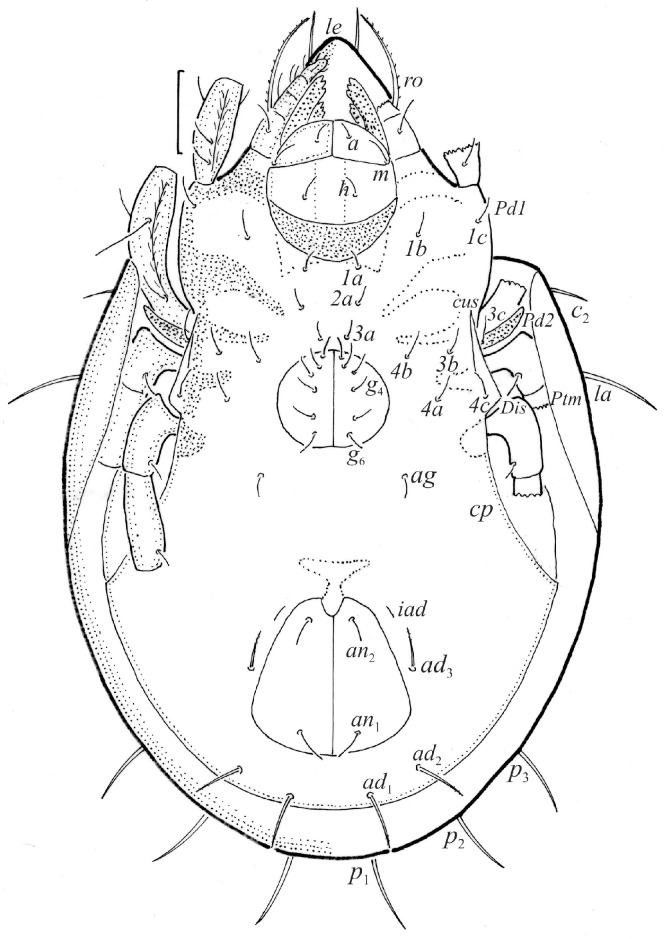
Adult female *Fuscozetes fuscipes*, ventral aspect, with legs partially drawn; scale bar: 50 μm.

**Figure 3:**
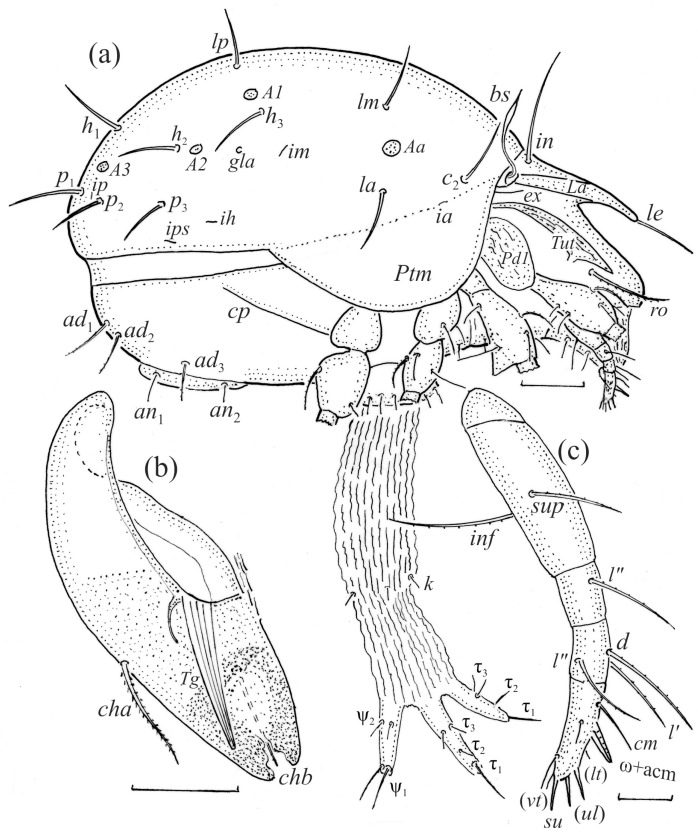
*Fuscozetes fuscipes*. (**a**) Female with ejected ovipositor, lateral aspect, with legs partially drawn; scale bar: 50 μm. Mouthparts of adult, right side; scale bars: 20 μm. (**b**) Chelicera, paraxial aspect. (**c**) Palp.

**Figure 4:**
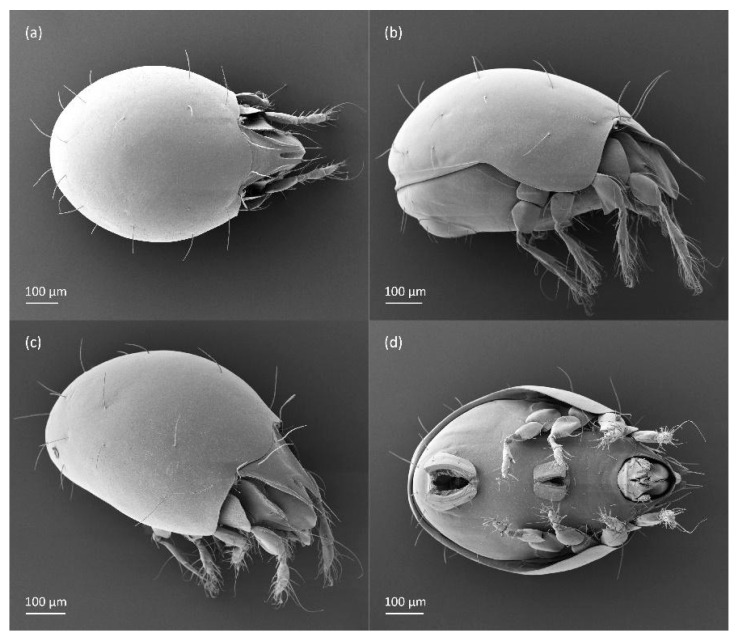
SEM micrographs of adult *Fuscozetes fuscipes*. (**a**) Dorsal view, (**b**) lateral view, (**c**) dorsolateral view, and (**d**) ventral view.

**Figure 5:**
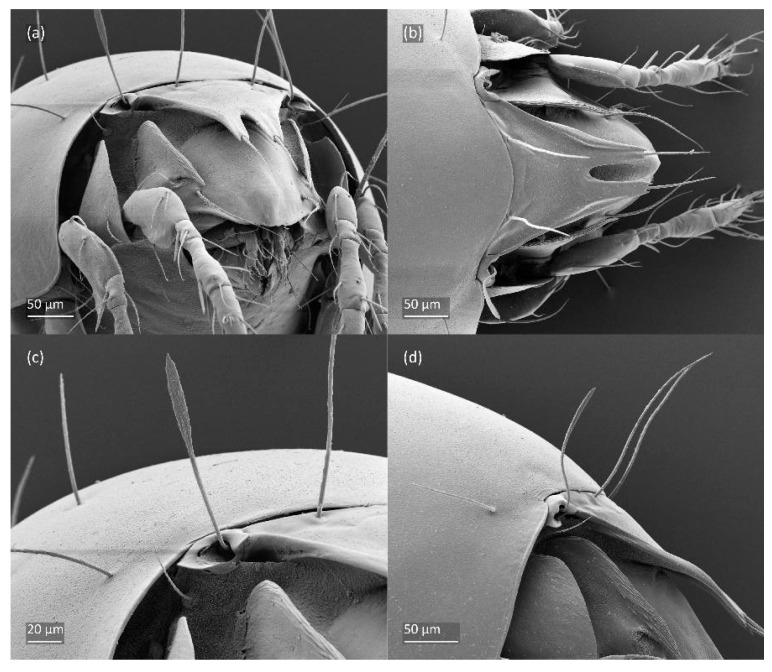
SEM micrographs of adult *Fuscozetes fuscipes*. Anterior part of body, (**a**) frontal view and (**b**) dorsal view; bothridial seta, (**c**) frontal view and (**d**) lateral view.

**Figure 6:**
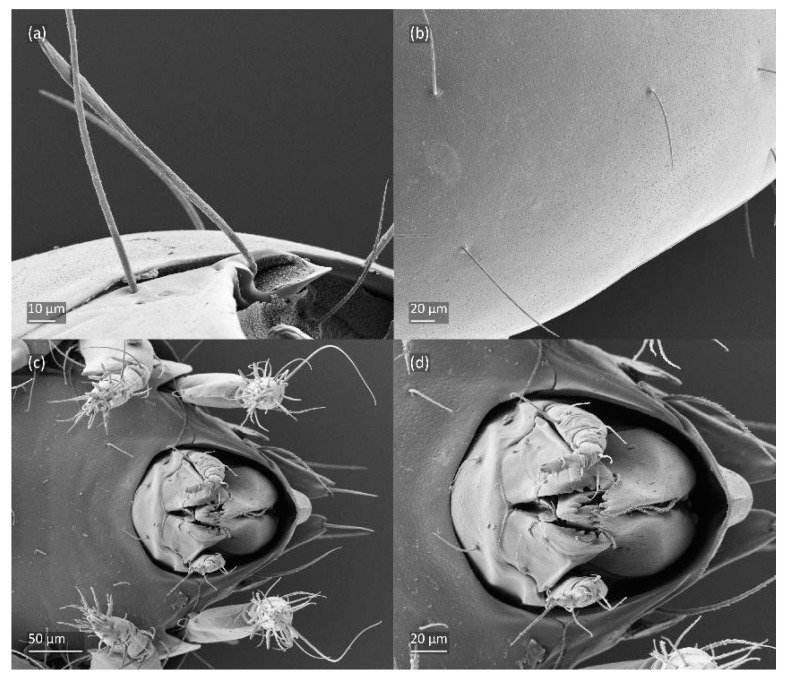
SEM micrographs of adult *Fuscozetes fuscipes*. (**a**) Bothridial seta, lateral view; (**b**) part of notogaster, dorsal view; and (**c**,**d**) anterior part of body, ventral view.

**Figure 7:**
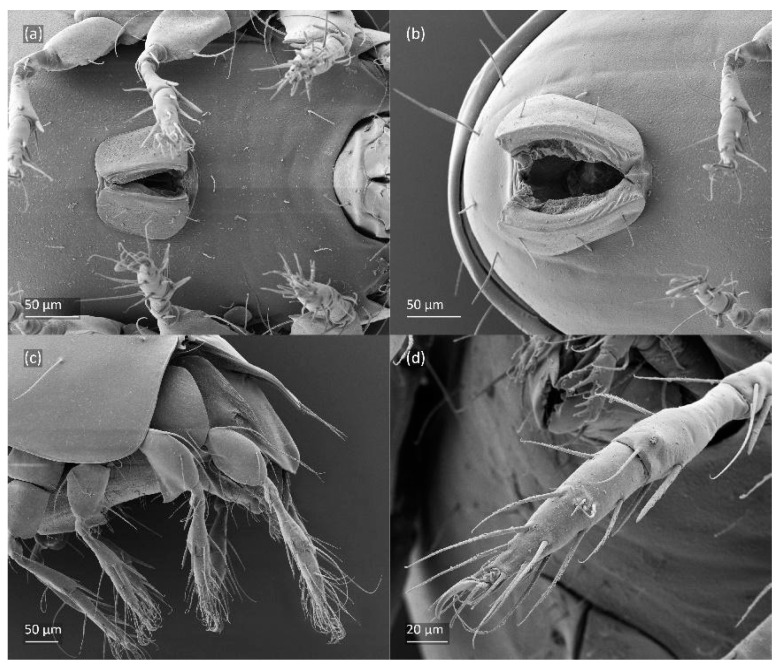
SEM micrographs of adult *Fuscozetes fuscipes*. Ventral view of (**a**) medial part of body and (**b**) posterior part of body; (**c**) anterior part of body, lateral view; and (**d**) part of leg I, dorsal view.

**Figure 8:**
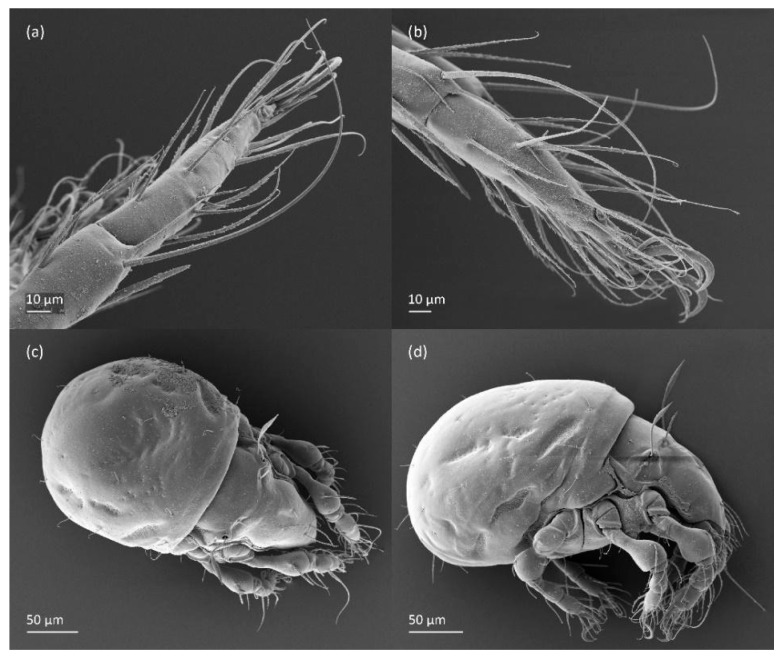
SEM micrographs of *Fuscozetes fuscipes*. Part of leg I of adult, (**a**) dorsal view and (**b**) lateral view; larva, (**c**) dorsal view and (**d**) lateral view.

**Figure 9:**
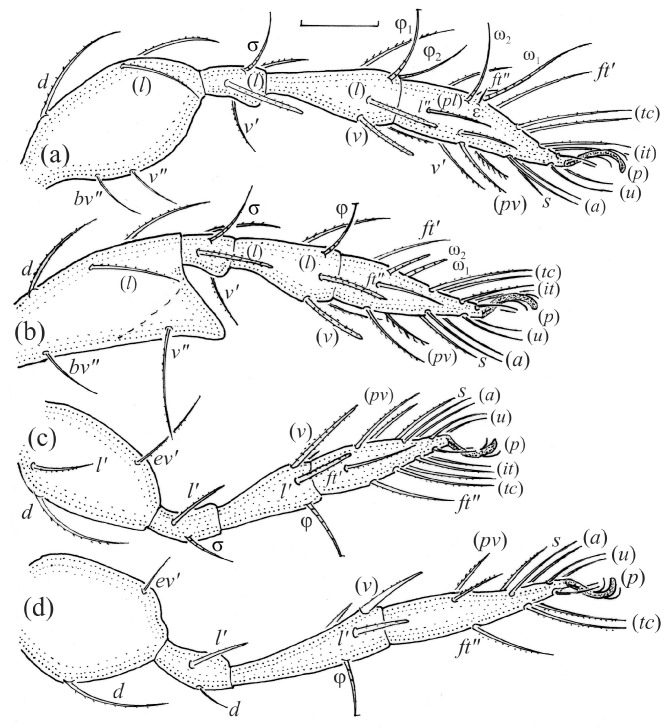
Leg segments of adult *Fuscozetes fuscipes* (femur to tarsus), right side; scale bar: 20 μm. (**a**) Leg I, (**b**) leg II, (**c**) leg III, and (**d**) leg IV.

The adults are brown to dark brown, oval in the dorsal and ventral view (Figure 1a, Figure 2 and Figure 4a,d), and of a medium size (734–897), as redescribed by Shaldybina [8] and Bayartogtokh and Weigmann [46] (but see Remarks). The mean length (and range) of females is 847.6 ± 19.7 (815–897, *n* = 23) and the maximum width is 566.2 ± 19.8 (538–587); the mean length (and range) of males is 791.7 ± 26.4 (734–815, *n* = 7) and the maximum width is 526.3 ± 20.4 (505–554). The prodorsal seta *in* is long, *le* and *ro* are of a medium size, and *ex* is short (Figure 1a, Figure 2, Figure 3a, Figure 4, Figure 5, Figure 6a and Figure 7c, Table 2). The notogastral setae (13 pairs, including *c*_2_) are long, with short barbs (Figure 1b and Figure 6a), but appear smooth under low magnification. The porose areas (four pairs) are rounded and *Aa* is slightly larger than the other porose areas (Figure 1a and Figure 3a). The chelicerae are chelate–dentate (Figure 3b and Figure 6c,d), with seta *cha* longer than *chb*; both setae are barbed. Most palp setae have short barbs (Figure 3c, Figure 5a and Figure 6c,d). The custodium (*cus*) is long and the discidium (*Dis*) is triangular. The epimeral setae are short and smooth, and the inner setae are slightly shorter than the other setae (Figure 2, Figure 4d, Figure 6c,d and Figure 7a,b). The genital (*g*), aggenital (*ag*), and anal (*an*) setae are short and smooth, and the adanal setae (*ad*) are longer, with short barbs on the apical part. The medium parts of femora I and II are oval in cross-section, without a large ventral carina, but femur II has an anteroventral projection (Figure 4b, Figure 5a, Figure 7c and Figure 8). Most of the leg setae have short barbs (Figure 4, Figure 5a,b, Figure 6c,d, Figure 7, Figure 8 and Figure 9a,b). The formulae of the leg setae [trochanter to tarsus (+ solenidia)] are as follows: I—1-5-3(1)-4(2)-20(2); II—1-5-3(1)-4(1)-15(2); III—2-3-1(1)-3(1)-15; and IV—1-2-2-3(1)-12.

Remarks: The adults investigated here were larger than those studied by Willmann [32] (body size: 710 × 500), Shaldybina [8] (body size: 753–774 × 473–516), Bayartogtokh and Weigmann [46] (body size: 629–676 × 419–433), and Weigmann [15] (body length of 630–765), but the length and distribution of the notogastral setae were similar in all studies.

**Figure 10:**
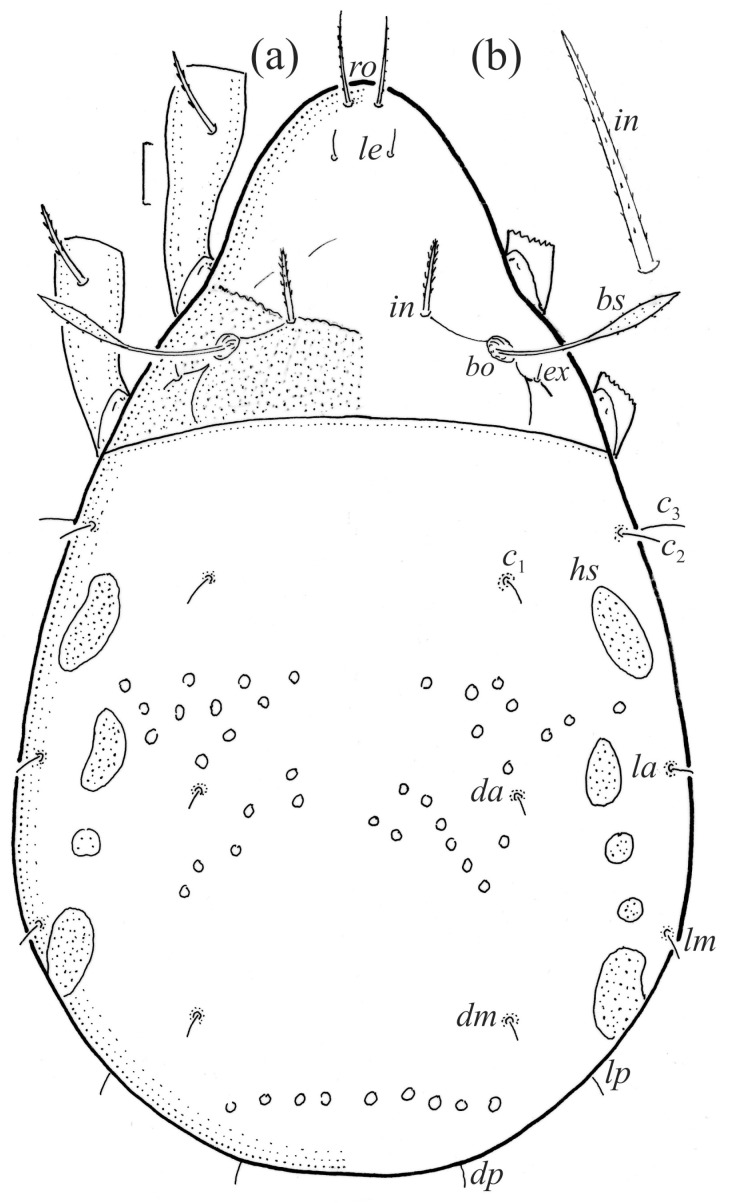
*Fuscozetes fuscipes* larva. (**a**) Dorsal aspect, legs partially drawn; scale bar: 20 μm. (**b**) Seta *in* (enlarged).

**Figure 11:**
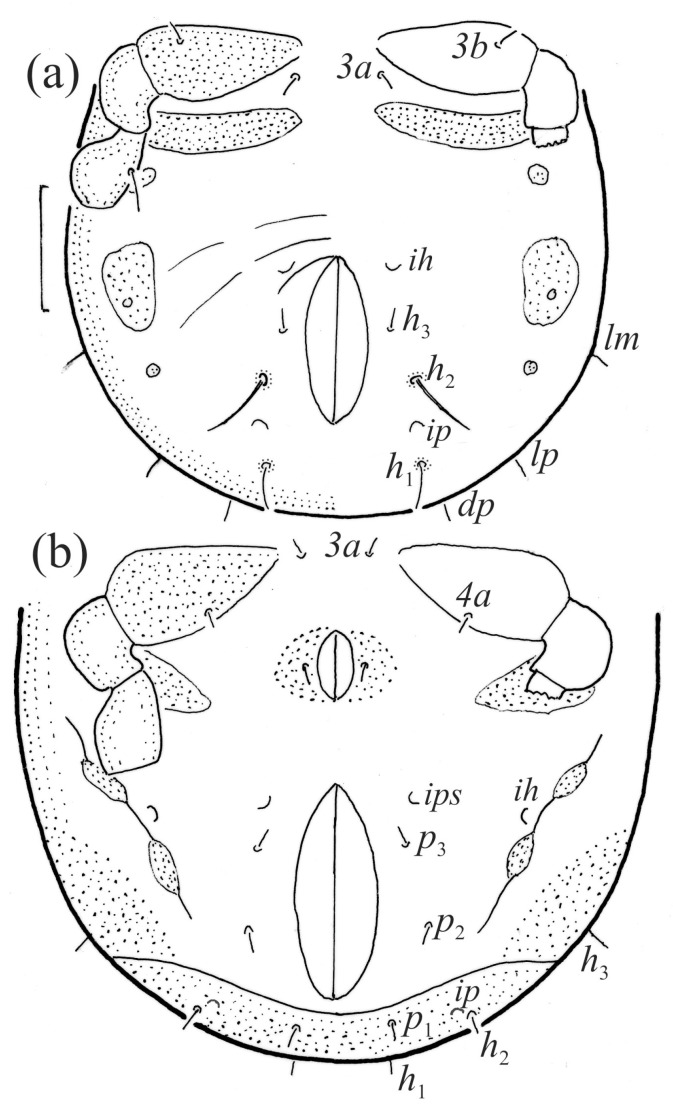
Posterior part of *Fuscozetes fuscipes* hysterosoma, legs III and IV partially drawn; scale bar: 20 μm. (**a**) Larva and (**b**) protonymph.

**Figure 12:**
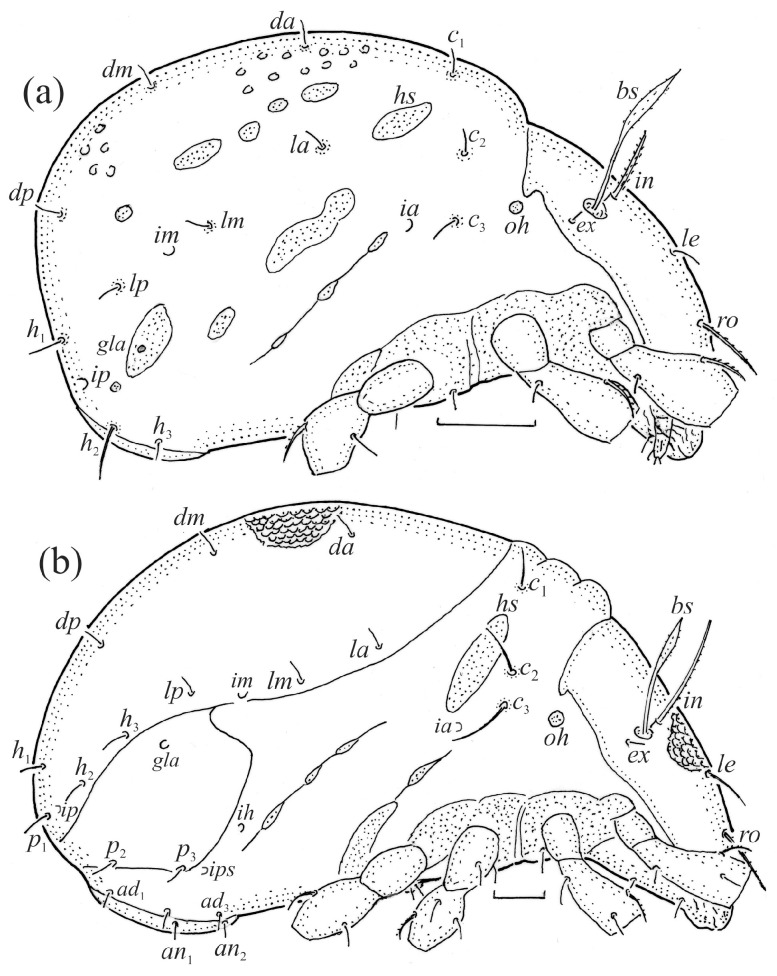
*Fuscozetes fuscipes*, lateral aspect, legs partially drawn; scale bars: 50 μm. (**a**) Larva and (**b**) tritonymph.

**Figure 13:**
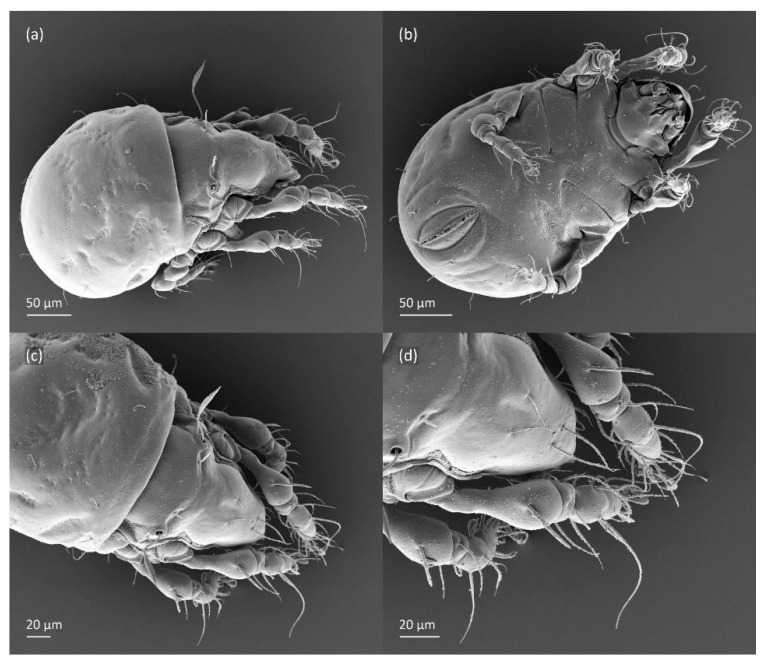
SEM micrographs of *Fuscozetes fuscipes* larva. (**a**) Dorsolateral view; (**b**) ventral view; and dorsal view of (**c**) anterior and medial part of body and (**d**) anterior part of body.

**Figure 14:**
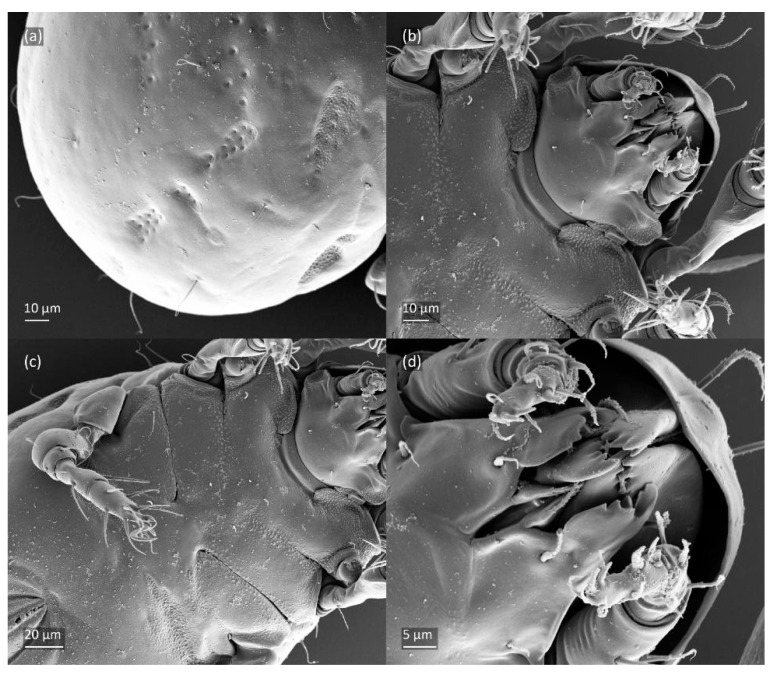
SEM micrographs of *Fuscozetes fuscipes* larva. (**a**) Posterior part of body, dorsolateral view; ventral view of (**b**) anterior part of body, (**c**) medial part of body, and (**d**) gnathosoma.

**Figure 15:**
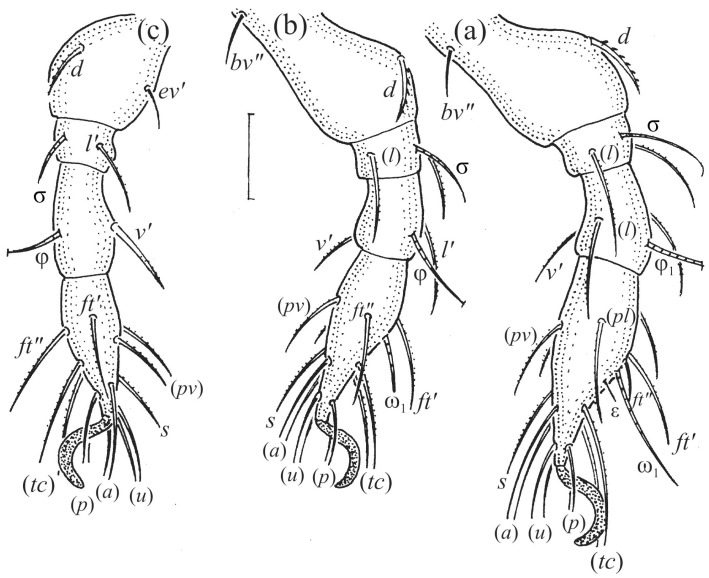
Leg segments of *Fuscozetes fuscipes* larva (femur to tarsus), right side; scale bar: 20 μm. (**a**) Leg I, (**b**) leg II, and (**c**) leg III.

**Figure 16:**
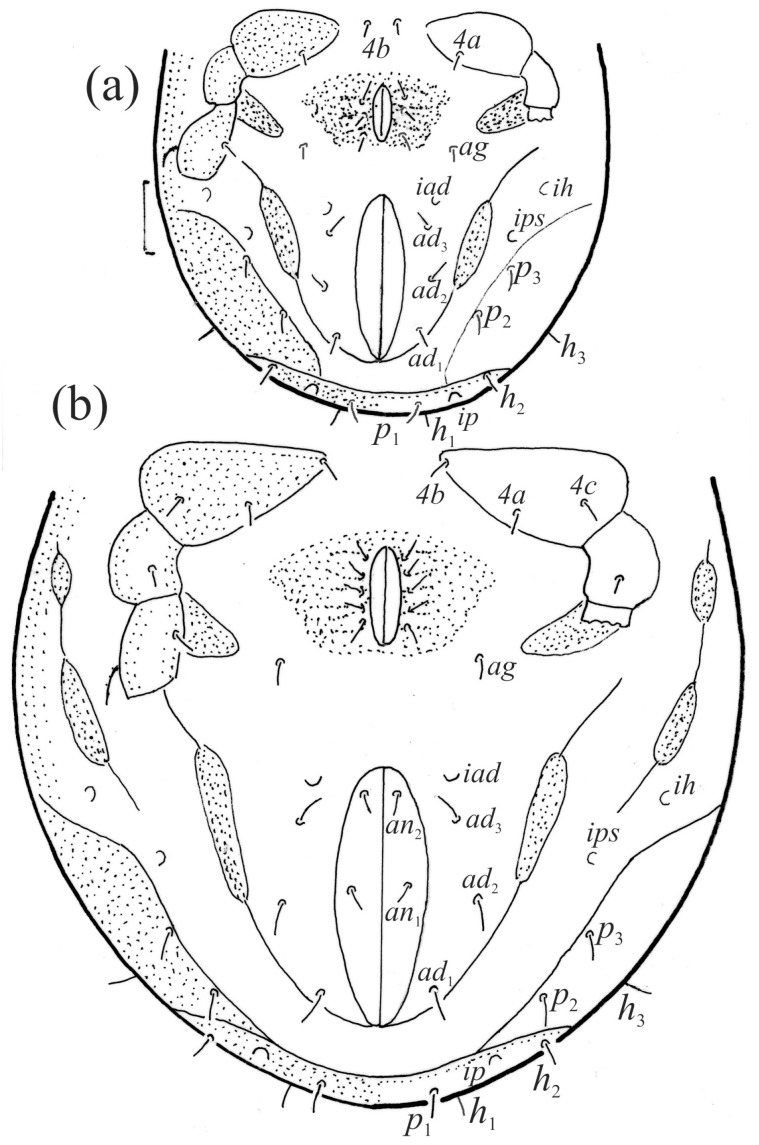
Anogenital region of *Fuscozetes fuscipes*, legs partially drawn; scale bar: 50 μm. (**a**) Deutonymph and (**b**) tritonymph.

**Figure 17:**
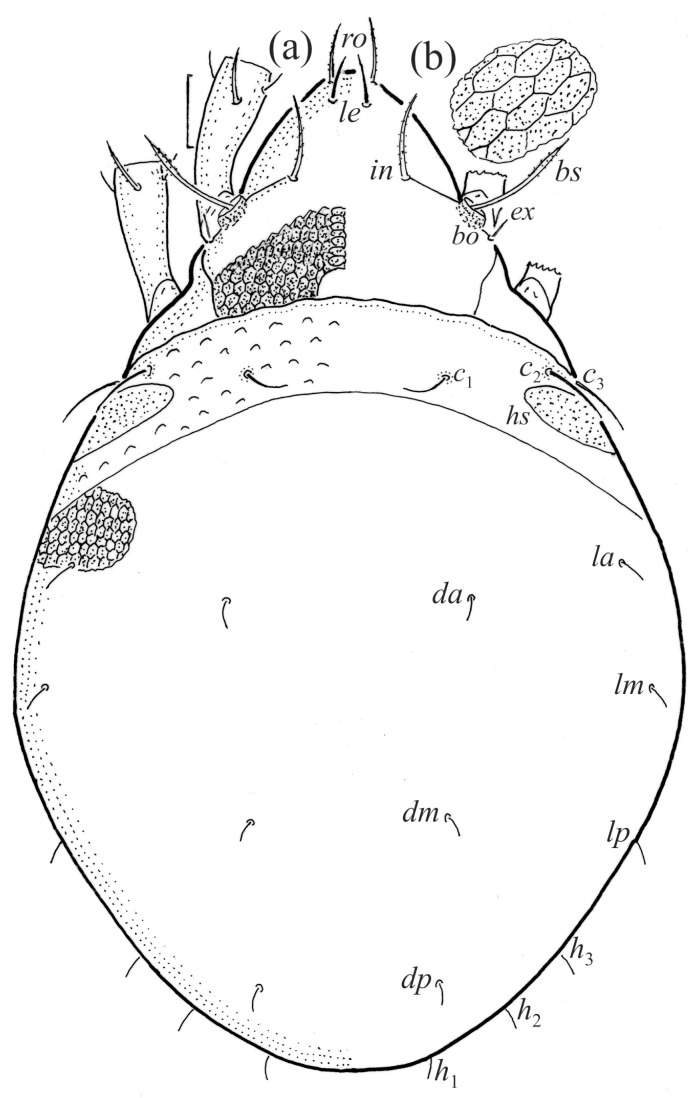
*Fuscozetes fuscipes* tritonymph. (**a**) Dorsal aspect, legs partially drawn; scale bar: 50 μm. (**b**) Pattern of gastronotum (enlarged).

**Figure 18:**
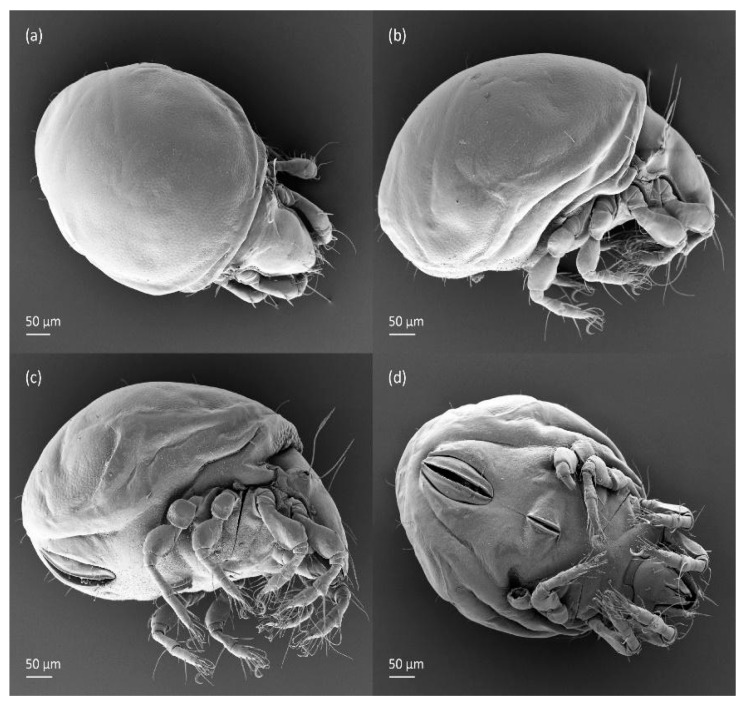
SEM micrographs of *Fuscozetes fuscipes* tritonymph. (**a**) Dorsal view, (**b**) lateral view, (**c**) ventrolateral view, and (**d**) ventral view.

**Figure 19:**
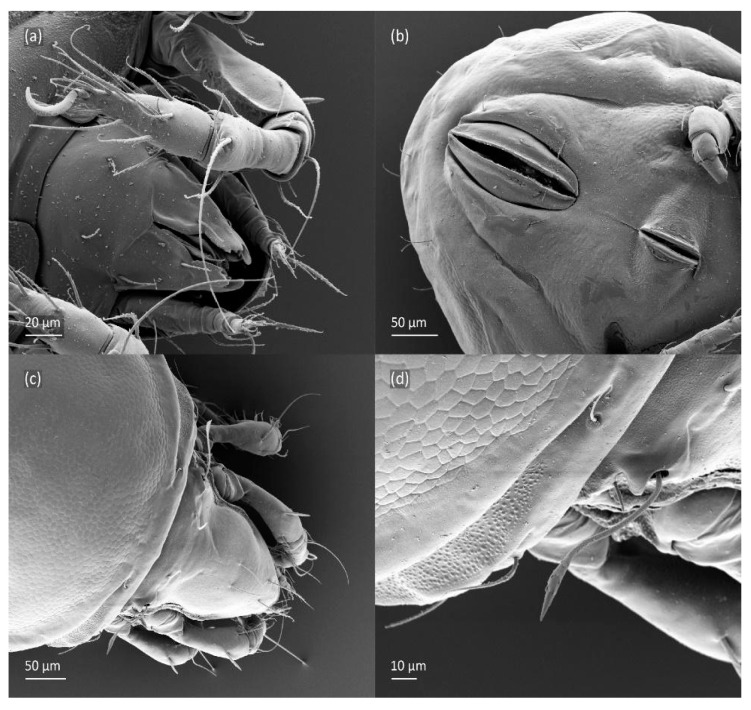
SEM micrographs of *Fuscozetes fuscipes* tritonymph. Ventral view of (**a**) anterior part of body and (**b**) posterior part of body; dorsal view of (**c**) anterior and medial part of body and (**d**) bothridial seta.

**Figure 20:**
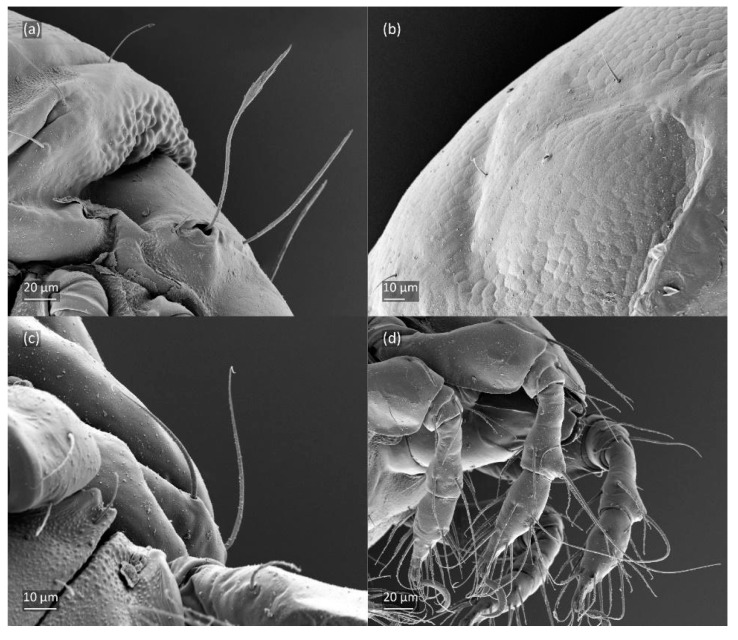
SEM micrographs of *Fuscozetes fuscipes* tritonymph. Lateral view of (**a**) bothridial seta and (**b**) *gla* opening; (**c**) seta *c*_2_ and *c*_3_, ventral view; and (**d**) legs I and II, lateral view.

**Figure 21:**
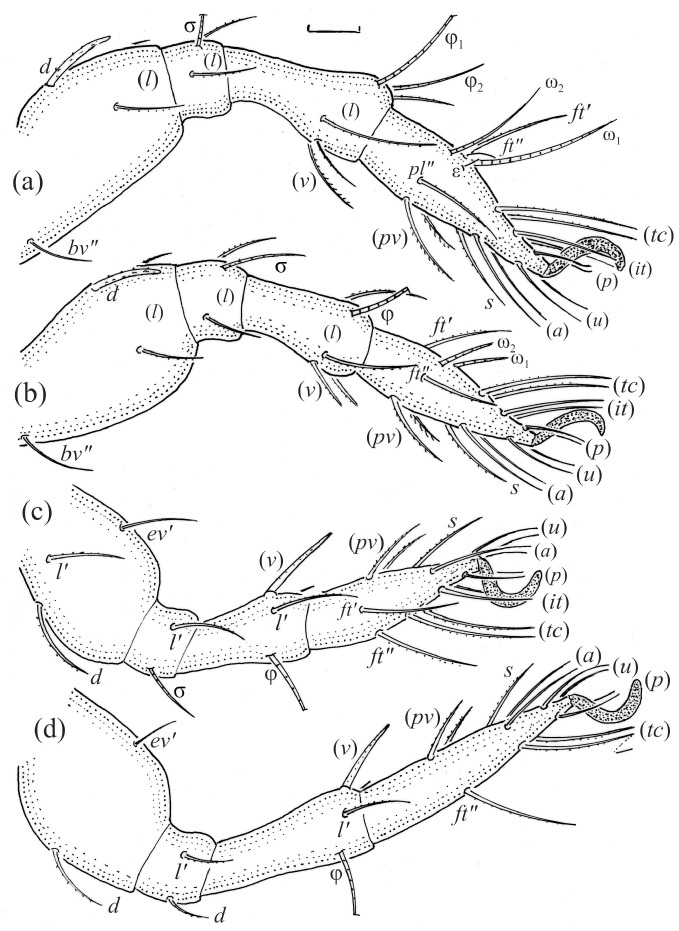
Leg segments of *Fuscozetes fuscipes* tritonymph (femur to tarsus), right side; scale bars: 20 μm. (**a**) Leg I, tarsus (*pl’* not illustrated); (**b**) leg II; (**c**) leg III; and (**d**) leg IV.

According to Shaldybina [8] and Weigmann [15], the porose area *Aa* was found to be larger than in the adults investigated herein and as reported by Bayartogtokh [47], which may reflect regional variation in this species. Shaldybina [8] drew dark areas around the notogastral setae, which are only observed in young, light-brown adults.

#### 3.2.3. Redescription of Juveniles

The larva is egg-shaped in its dorsal and ventral view (Figure 9c, Figure 10a, Figure 11a and Figure 13b), and its body is light brown with a darker prodorsum, sclerites, epimeres, and legs. The prodorsum is subtriangular, the rostrum is rounded, setae *ro* and *in* are of a medium size and finely barbed, and the other setae are short and smooth (Figure 9c,d, Figure 10a, Figure 12a, Figure 13 and Table 2). The mutual distance between setal pair *le* is almost two times longer than that between setae *ro*, and the mutual distance between pair *in* is about four times longer than that between pair *ro*. Setal pair *le* is placed approximately midway between the setal pairs *in* and *ro*. The opening of the bothridium is rounded and the bothridial seta is fusiform, with a barbed head. A ridge is present between the opening of the bothridium and the insertion of seta *in*. The prodorsum is finely porose.

The gastronotum of the larva has 12 pairs of setae, including *h*_3_ inserted laterally to the anterior part of the anal valves (Figure 9d, Figure 11a, Figure 12a and Figure 13b). The gastronotal shield is poorly developed and most of the gastronotal setae are short and smooth (Figure 9c,d, Figure 10a, Figure 11a, Figure 12a, Figure 13a–c and Figure 14a,c), except for a slightly longer *h*_2_. Most of the setae are located on basal microsclerites except for *h*_3_. The humeral organ is rounded and located anteriorly to seta *c*_3_. The humeral sclerite is oval and porose, three or four other macrosclerites are present laterally to setae *da* and *dm*, and many microsclerites are present in the central and posterior parts of the gastronotum. Three large macrosclerites are present on the lateral side of the gastronotum, including one around the *gla* opening, along with 3–4 small sclerites (Figure 10a, Figure 11a and Figure 12a). A large sclerite is present posteriorly to leg III. Cupule *ia* is located posteriorly to seta *c*_3_, cupule *im* is located posteriorly to seta *lm*, *im* is located between setae *h*_1_ and *h*_2_, and *ih* is lateral to the anterior end of the anal opening (Figure 10a, Figure 11a and Figure 12a). The gland opening *gla* is located laterally to seta *lp*. The paraproctal valves (segment PS) are glabrous. The chelicera is chelate–dentate (Figure 14b,d). All the femora are oval in cross-section, a large ventral carina is absent, and most leg setae are finely barbed (Figure 9c,d, Figure 13, Figure 14b–d and Figure 15).

The shape and color of the protonymph and other nymphs are the same as in the larva, but the head of the bothridial seta is slimmer, and the prodorsum and gastronotal shield have a reticulate cuticle (Figure 12b, Figure 17, Figure 18, Figure 19b–d and Figure 20a,b). The gastronotum has 15 pairs of setae because the setae of the *p*-series appear in the protonymph and are retained in subsequent nymphs; all of these setae are short and smooth except for the medium-sized *c*-series setae (Figure 11b, Figure 12b, Figure 16, Figure 17, Figure 18, Figure 19b–d, Figure 20a–c and Table 2). The humeral organ is located in the same location as in the larva, the humeral sclerite has seta *c*_1_, and the other setae of the *c*-series are located on the basal microsclerites. The gastronotal shield is well developed, with 10 pairs of setae (*d*-, *l*-, and *h*-series, and *p*_1_), and setae *p*_2_ and *p*_3_ are placed on a large posteroventral sclerite. Small sclerites are present laterally to the setae of the *l*-series, and a large macrosclerite is present posteriorly to leg IV (Figure 11b, Figure 12b and Figure 16). In the protonymph, one pair of genital setae is present on the genital valves, and two pairs are added in both the deutonymph and the tritonymph (Figure 11b, Figure 16, Figure 18d and Figure 19b). The genital valves are placed on a large macrosclerite. In the deutonymph, one pair of aggenital setae and three pairs of adanal setae appear, and they remain in the other nymphs; all are short and smooth. In the tritonymph, cupules *ia* and *im* are placed in the same manner as in the larva, cupule *ip* is between setae *p*_1_ and *h*_2_, cupule *iad* is lateral to the anterior part of the anal valves, and cupules *ih* and *ips* are pushed laterally to cupule *iad* (Figure 11b, Figure 12b and Figure 16). The gland opening *gla* is placed anterolaterally to seta *h*_3_. The chelicerae are chelate–dentate (Figure 18d and Figure 19a). The anal valves of the protonymph and deutonymph are glabrous, and those of the tritonymph have two pairs of short and smooth setae. All the femora are oval in cross-section, a large ventral carina is absent, and most of the leg setae are finely barbed (Figure 18, Figure 19, Figure 20d and Figure 21). In one deutonymph, two setae *v’* were present on trochanter III.

#### 3.2.4. Summary of Ontogenetic Transformations

In the larva, the prodorsal setae *ro* and *in* are of a medium size, and the setae *le* and *ex* are short, whereas in the nymphs and adult, seta *in* is clearly longer than *ro*, and seta *le* is of a medium size. In all the juveniles, the bothridium is rounded, whereas in the adult, it is larger and gains scales. In all the instars, the bothridial seta is fusiform with a barbed head, but in the nymphs and adult, the head is slimmer than in the larva. The larva has 12 pairs of gastronotal setae (*h*_3_ is present), while the nymphs have 15 pairs. The notogaster of the adult loses setae *c*_1_, *c*_3_, and those of the *d*-series, such that 10 pairs of setae remain on the notogaster. The formula of gastronotal setae in *F*. *fuscipes* is 12-15-15-15-10 (from larva to adult). The formulae of the epimeral setae are: 3-1-2 (larva), 3-1-2-1 (protonymph), 3-1-2-2 (deutonymph), and 3-1-3-3 (tritonymph and adult); the formula of the genital setae is 1-3-5-6 (protonymph to adult); the formula of the aggenital setae is 1-1-1 (deutonymph to adult); and the formula of segments PS–AN is 03333-0333-022. The ontogeny of the leg setae and solenidia of *F*. *fuscipes* is given in Table 3.

### 3.3. Mitochondrial Genetic Variation

**Figure 22:**
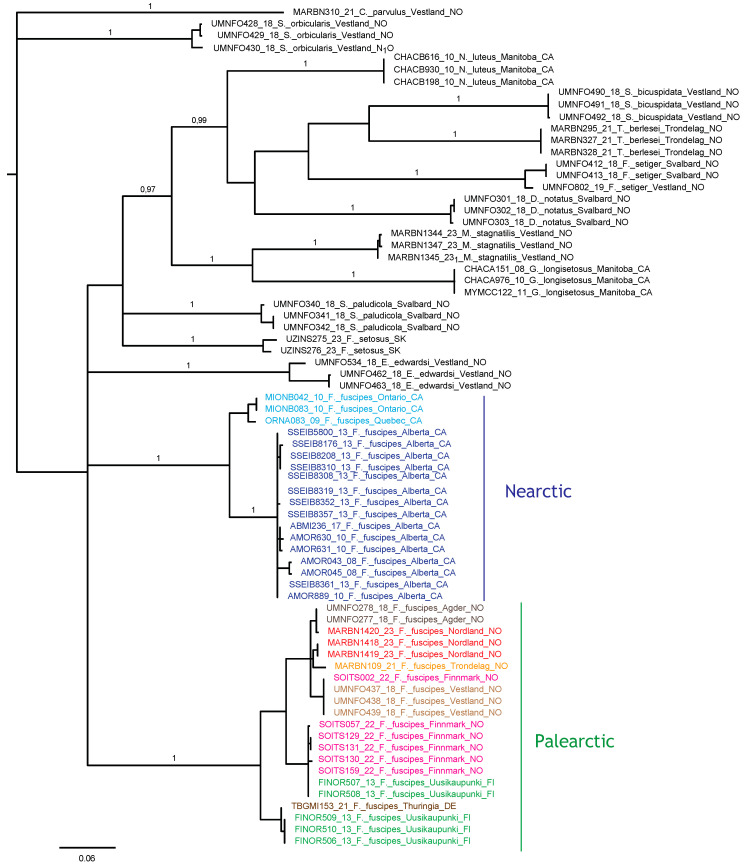
Bayesian tree topology based on COI nucleotide sequences (658 bp). Specimen numbers correspond to those in the BOLD database (http://boldsystems.org/, accessed on 20 November 2023). Information about barcoded specimens is presented in Table 1.

The Bayesian analysis of the COI sequences revealed two deeply diverged clades of *F. fuscipes* corresponding to exclusively Nearctic and Palearctic populations, respectively (Figure 22), and separated by a 15.5–18.4% uncorrected p-distance. Each regional clade contained 11 haplotypes, with a maximum of 7.2% divergence in the Nearctic clade and 5.8% in the Palearctic clade. Several Palearctic haplotypes were shared between distant locations, including Finnmark and Vestland in Norway, and one haplotype was shared between Finnmark and SW Finnland. Near-identical haplotypes were found in Agder and Nordland (Norway), and in SW Finland and Germany.

### 3.4. Ecology and Biology

We collected *F*. *fuscipes* in *Sphagnum* mosses on the shore of lake Skomakerdiket (Bergen, Norway), where this species achieved a density of 102 individuals per 500 cm^3^. In this population, the juveniles made up 52% of all individuals, with the following stage structure: 8 larvae, 33 protonymphs, 4 deutonymphs, 7 tritonymphs, and 50 adults. The female-to-male sex ratio was 1:0.3, and 7% of the females were gravid and carried one or two large eggs (290 × 175), comprising 34% of the length of the females.

## 4. Discussion

### 4.1. Morphology and Development

The juvenile stages of *F*. *fuscipes* from Norway are generally similar to those from Russia [48] and Poland [17], except for the shape of some setae and sclerites and the number of microsclerites in the larvae. In the larvae from Norway, the prodorsal seta *in* is of a medium size and barbed, as in the larvae from Poland [17], whereas in the larvae from Russia [48], this seta is short and smooth. In all regions, the larvae of *F*. *fuscipes* lack a gastronotal shield, most of the gastronotal setae are located on the microsclerites and other sclerites, and microsclerites are also present on the gastronotum. However, the shape of the macrosclerites differs in these larvae, and the larvae from Norway and Russia have more microsclerites than those from Poland. In the tritonymphs from Norway and Poland, the gastronotal setae are slightly longer than in the specimens from Russia. All these differences probably illustrate regional variation in the species.

Seniczak et al. [14] compared a selection of morphological characters in several *Fuscozetes* species. In light of this comparison and this investigation, the largest is *F*. *fuscipes*, the smallest is *F*. *setiger*, and the body length of most species overlaps. These species also differ from one another in the shape of their bothridial seta, translamella, lamellar cusp, and porose area *Aa*, and in the number and shape of their notogastral setae. Most species have 10 pairs of notogastral setae (*c*_2_ is present); two species (*F*. *novus* Shaldybina, 1969, and *F*. *tatricus*) have 11 pairs (*c*_2_ and *dp* are present), and *F*. *setosus* has 10–13 pairs of setae (*c*_2_ and some or all the setae of the *d*-series are present).

Seniczak et al. [14] also compared 23 morphological characters of the larvae and tritonymphs of *F*. *coulsoni*, *F*. *fuscipes*, *F*. *kamchatkicus*, *F*. *setiger*, *F*. *setosus*, and *F*. *tatricus*. The juveniles of *F*. *fuscipes* are the most similar to those of *F*. *setosus*, differing from them in six morphological characters, and the most dissimilar from those of *F*. *setiger*, differing from them in 21 morphological characters. This is not too surprising, in view of the distant phylogenetic relationship to *F. setiger* indicated by our COI data analysis (Figure 22).

The morphological ontogeny of *F*. *fuscipes* is generally similar to that of *F*. *coulsoni*, *F*. *kamchatkicus*, *F*. *pseudosetosus*, *F*. *setiger*, *F*. *setosus*, and *F*. *tatricus* [8,12,13,14,17,49,50,51], except for the shape of the larval gastronotum. In *F*. *fuscipes*, *F*. *pseudosetosus*, and *F*. *tatricus*, the gastronotal shield is absent, but macrosclerites and microsclerites can be present, whereas in the other species, the gastronotal shield and a humeral macrosclerite are present, and sometimes other macrosclerites and microsclerites. Among these species, the ontogeny of the leg setae and solenidia were investigated in detail in *F*. *coulsoni*, *F*. *kamchatkicus*, and *F*. *setiger* [12,13,14]. The ontogeny of the leg setae and solenidia of *F*. *fuscipes* studied herein is most similar to that of *F*. *setiger*, differing from it in two morphological characters, and most dissimilar to that of *F*. *kamchatkicus*, differing from it in five morphological characters (Table 4). Some leg characters can be diagnostic.

The morphology of the adults and juveniles of *Fuscozetes* is generally similar to those of *Melanozetes* [9,10,11,52,53,54,55,56,57,58,59,60], except for the diagnostic characters for these genera. The adults of *Fuscozetes* have 10–13 pairs of notogastral setae, including *c*_2_ and some or all the setae of the *d*-series, whereas those of *Melanozetes* have 14 pairs of setae, including *c*_2_ and *c*_3_ [11,56,57,58,59].

The separation of the juveniles of *Fuscozetes* from those of *Melanozetes* is more difficult than the adults. The juveniles of *Fuscozetes* have a generally smaller area of sclerites on the gastronotum that those of *Melanozetes*, except for the larvae of some species. For example, the larva of *Melanozetes avachai* Seniczak et al. 2016 [58] has a weakly developed gastronotal shield, most of its gastronotal setae are located on microsclerites, femora I and II are oval in cross-section, and a large ventral carina is absent, as with that of *F*. *fuscipes* studied herein. The separation of the nymphs and adults of *Fuscozetes* from those of *Melanozetes* is easier than for the larva, mainly using the length and location of solenidion ω_2_ on tarsus I; in *Fuscozetes*, this solenidion is shorter than ω_1_ and is placed posterolaterally to ω_1_, whereas in *Melanozetes*, solenidion ω_2_ is as long as or longer than ω_1_ and is placed anteriorly to ω_1_.

The three-dimensional SEM figures of *F*. *fuscipes* correspond well with the line drawings of this species, which are, to some degree, subjective and depend on the technique of preparation and the author. For example, in the larva of *F*. *fuscipes*, some macrosclerites and microsclerites are observed in different aspects as depressions, which are rarely observed in SEM figures. In the tritonymph, the reticulation of the gastronotum and a humeral sclerite are well observed.

### 4.2. Ecology and Distribution

*Fuscozetes fuscipes* has a Holarctic distribution [1]. In Norway, it has been found in moist mosses in the north (Finnmark), west (Vestland), and south [60,61,62,63]. *Fuscozetes fuscipes* is a hygrophilous [64,65] or meso-hygrophilous species [66,67]. It inhabits wet tundra [10], *Sphagnum* mosses, and wet habitats [65,68,69,70] close to pools and lakes [71,72,73,74], as well as wet-to-humid forest soils and meadows [45]. In an oligotrophic bog in Norway, it was found only in the lower *Sphagnum* layer, 10–17 cm deep [63,75]. Solhøy Wunderle and Solhøy [76] consider *F*. *fuscipes* a true arctic and high-mountain species, and Schatz [77] and Murvanidze et al. [45] confirmed the dominance of this species in subalpine zones, while Solhøy found it in an alpine zone in Norway [75]. This species is sensitive to some pesticides [78], but it is cold-tolerant and able to survive winter temperatures of −28 °C [79].

Wallwork [80] investigated various biological aspects of *F*. *fuscipes* in the laboratory. Adults and nymphs were fed on the macerated leaf tissue of hemlock (*Conium maculatum* L.) and on the moist decaying leaves and petioles of yellow birch (*Betula alleghaniensis* Britt.), and the nymphs also fed on dead mites and springtails. Madge [65] observed no clear response by adults and juveniles to a higher air humidity in similar experiments, whereas in dry air, these wet-adapted mites quickly died. Nevertheless, this species survives much longer in a lower humidity than another hygrophilous species, *H*. *rufulus* C.L. Koch, 1835, probably due to its thicker waxy cuticle on the body [66,81].

Shaldybina [82] cultivated *F*. *fuscipes* under laboratory conditions at 18–20 °C; fed it on lichens, mosses, and raw potatoes; and estimated a development time of 86 days for this species. The mean time of the development of successive instars (+ immobile period between stages) was in days: egg, 12; larva, 11.5 + 4; protonymph, 12 + 4; deutonymph, 14.5 + 4; and tritonymph, 17.5 + 6.5. Among the 18 species cultivated by this author, the time of development varied between 40 and 180 days. Under natural conditions in cooler climates, the time of the development of *F*. *fuscipes* probably lasts longer than 86 days, which limits its population growth. In our investigation, only 7% of the females of this species were gravid, each carried one or two large eggs, and in June, the number of juveniles was approximately similar to that of the adults. In earlier studies carried out in a similar area at two lake shores, also in June, the juveniles were more abundant than the adults and made up 71% and 81% of each local population [83].

There are no specific data on the dispersal of *F*. *fuscipes*, but it can probably use many passive ways of spreading that are common in oribatid mites. Many oribatids can migrate over large distances with the wind (anemohydrochory) [84,85,86,87], via birds [88,89,90,91,92], by water currents or waves (together with the action of wind), or with objects drifting in water [84,86,93], including transport in seawater [94]. Even though Oribatida lack obvious morphological adaptations for active transport by phoresy, it has been demonstrated that they are carried on insects [95,96,97,98,99,100] and frogs [101].

The several shared or near-identical haplotypes between distant sites sampled in this study indicate high migration rates of this species in Europe, at least in modern times. A similar pattern of haplotype identity was found for *Platynothrus peltifer* (C.L. Koch, 1839) in Western Norway, Belgium, and Germany [102]; *P. punctatus* (L. Koch, 1879) in Svalbard, Western Norway, and Southern Spain [103]; and *Nanhermannia coronata* Berlese, 1913, in Northern, Central, and Southern Norway, Ireland, and Finland [104]. We may hypothesize that their migration in a latitudinal direction is largely influenced by migrating birds. In contrast, the longitudinal separation of subclades in Canada, and between Canada and Scandinavia, in two different clades of *F*. *fuscipes* indicates much less migration in this direction. The morphology of *F*. *fuscipes* from Canada has not been studied in detail, including the juvenile stages, so it is not possible to pinpoint any morphological differences between the Nearctic and Palearctic populations. To enable a firm test of species boundaries and taxon validity, more genetic data and morphological studies are needed, because a single and rapidly evolving mitochondrial marker is not sufficiently informative to conclude on such issues.

Furthermore, we note that other oribatid genera have mixed patterns of genetic differentiation across the Atlantic, e.g., *Platynothrus troendelagicus* Seniczak and Seniczak, 2022, which has identical COI haplotypes across Europe (Norway and Ireland) and Canada [105], whereas populations of *P. peltifer* have diverged significantly between Europe, USA, and Japan. It is, therefore, possible that more intensive sampling of *F*. *fuscipes* will support a similar cryptic-species scenario. In this context, it will be useful to study the ecological traits that may affect long-distance colonization in this species group.

## 5. Conclusions

1. *Fuscozetes* species are a well-formed morphological group of mites that differ clearly from closely related *Melanozetes* species, both as nymphs and adults. The adults of *Fuscozetes* have fewer notogastral setae (10–13 pairs, including *c*_2_ and some or all the setae of the *d*-series) than those of *Melanozetes* (14 pairs, including *c*_2_ and *c*_3_), and the adults and nymphs have a shorter solenidion ω_2_ on tarsus I than ω_1_, and it is placed posterolaterally to ω_1_. In *Melanozetes*, solenidion ω_2_ is as long as or longer than ω_1_ and is placed anteriorly to ω_1_.
2. *Fuscozetes fuscipes* is a hygrophilous species and prefers wet tundra, *Sphagnum* mosses, and wet habitats close to pools and lakes.
3. Mitochondrial genetic data revealed deeply diverged populations across the Holarctic, a high local and regional genetic diversity, and several examples of haplotypes shared between distant Scandinavian localities, indicating latitudinal long-distance migration.

## Figures and Tables

**Table 1:** Information about sequenced specimens of *Fuscozetes fuscipes* and other oribatid species used in this study. Na—not available; these sequences are public in BOLD, but without some data.

Species	Sequence Code at BOLD	GenBank Access No.	Locality	Coordinates	Elevation m a. s. l.	Reference
*Fuscozetes fuscipes* (C.L Koch, 1844)	MARBN1420-23	PP215015	NO: Nordland	68.292, 14.186	60	-
	MARBN1419-23	PP214995	NO: Nordland	68.292, 14.186	60	-
	MARBN1418-23	PP215016	NO: Nordland	68.292, 14.186	60	-
	SOITS159-22	PP215024	NO: Finnmark	69.713, 30.871	40	-
	SOITS131-22	PP214998	NO: Finnmark	69.713, 30.871	40	-
	SOITS130-22	PP214996	NO: Finnmark	69.713, 30.871	40	-
	SOITS129-22	PP215013	NO: Finnmark	69.713, 30.871	40	-
	SOITS057-22	PP215003	NO: Finnmark	69.143, 29.240	61	-
	SOITS002-22	PP215011	NO: Finnmark	69.143, 29.240	61	-
	MARBN109-21	PP215006	NO: Trøndelag	63.489, 8.874	50	-
	UMNFO278-18	PP215005	NO: Agder	58.451, 8.705	62	-
	UMNFO439-18	PP215021	NO: Vestland	60.398, 5.351	370	-
	UMNFO438-18	PP215026	NO: Vestland	60.398, 5.351	370	-
	UMNFO437-18	PP215027	NO: Vestland	60.398, 5.351	370	-
	UMNFO277-18	MN520688	NO: Agder	58.451, 8.705	62	Seniczak et al. [25]
	ABMI236-17	MN348906	CA: Alberta	53.506, −114.960	780	Young et al. [26]
	ABMI237-17	MN355322	CA: Alberta	51.734, −113.641	904	Young et al. [26]
	AMOR043-08	MN348553	CA: Alberta	51.847, −114.764	1054	Young et al. [26]
	AMOR045-08	MN354723	CA: Alberta	51.847, −114.764	1054	Young et al. [26]
	AMOR630-10	MN354116	CA: Alberta	53.533, −113.533	780	Young et al. [26]
	AMOR631-10	MN351373	CA: Alberta	53.533, −113.533	780	Young et al. [26]
	AMOR889-10	MN350498	CA: Alberta	53.208, −115.651	780	Young et al. [26]
	MIONB042-10	HM887577	CA: Ontario	45.390, −78.906	386	Young et al. [26]
	MIONB083-10	HQ575095	CA: Ontario	45.390, −78.906	386	Young et al. [26]
	ORNA083-09	MN356812	CA: Quebec	45.610, −76.004	176	Young et al. [26]
	SSEIB5800-13	KM828928	CA: Alberta	53.567, −112.851	722	Young et al. [26]
	SSEIB8176-13	KM834684	CA: Alberta	53.567, −112.851	722	Young et al. [26]
	SSEIB8208-13	KM834777	CA: Alberta	53.567, −112.851	722	Young et al. [26]
	SSEIB8308-13	KM840220	CA: Alberta	53.567, −112.851	722	Young et al. [26]
	SSEIB8310-13	KM840191	CA: Alberta	53.567, −112.851	722	Young et al. [26]
	SSEIB8319-13	KM828000	CA: Alberta	53.567, −112.851	722	Young et al. [26]
	SSEIB8352-13	KM831118	CA: Alberta	53.567, −112.851	722	Young et al. [26]
	SSEIB8357-13	KM824343	CA: Alberta	53.567, −112.851	722	Young et al. [26]
	SSEIB8361-13	KM833473	CA: Alberta	53.567, −112.851	722	Young et al. [26]
	FINOR506-13	MZ623348	FI: Uusikaupunki	60.814, 21.216	12	Roslin et al. [27]
	FINOR507-13	MZ623712	FI: Uusikaupunki	60.814, 21.216	12	Roslin et al. [27]
	FINOR508-13	MZ626462	FI: Uusikaupunki	60.814, 21.216	12	Roslin et al. [27]
	FINOR509-13	MZ628127	FI: Uusikaupunki	60.814, 21.216	12	Roslin et al. [27]
	FINOR510-13	MZ626956	FI: Uusikaupunki	60.814, 21.216	12	Roslin et al. [27]
	TBGMI153-21	Na	GE: Thuringia	51.083, 10.426	447	-
*Ghilarovizetes longisetosus* (Hammer, 1952)	CHACA151-08	JX835704	CA: Manitoba	58.760, −94.069	29	Young et al. [26,28]
	CHACA976-10	HM405840	CA: Manitoba	58.786, −93.739	281	Young et al. [26,28]
	MYMCC122-11	JX834086	CA: Manitoba	58.771, −93.851	281	Young et al. [26,28]
*Neogymnobates luteus* Hammer, 1955	CHACB198-10	HQ558468	CA: Manitoba	58.731, −93.781	281	Young et al. [26,28]
	CHACB616-10	HQ558703	CA: Manitoba	58.623, −94.230	8	Young et al. [26,28]
	CHACB930-10	HM907357	CA: Manitoba	58.625, −93.816	38	Young et al. [26,28]
*Ceratozetes parvulus* Sellnick, 1922	MARBN125-21	PP215014	NO: Trøndelag	60.593, 7.432	1166	-
	MARBN310-21	PP215030	NO: Vestland	60.593, 7.432	1166	-
	MARBN311-21	PP215035	NO: Vestland	60.593, 7.432	1166	-
*Melanozetes stagnatilis* (Hull, 1914)	MARBN1344-23	PP215020	NO: Vestland	60.794, 5.055	46	-
	MARBN1345-23	PP215008	NO: Vestland	60.794, 5.055	46	-
	MARBN1347-23	PP215022	NO: Vestland	60.794, 5.055	46	-
*Trichoribates berlesei* (Jacot, 1929)	MARBN295-21	PP215033	NO: Trøndelag	63.405, 10.120	113	-
	MARBN327-21	PP215032	NO: Trøndelag	63.405, 10.120	113	-
	MARBN328-21	PP214999	NO: Trøndelag	63.405, 10.120	113	-
*Diapterobates notatus* (Thorell, 1871)	UMNFO301-18	PP215034	NO: Svalbard	78.2037, 15.319	161	-
	UMNFO302-18	PP215025	NO: Svalbard	78.2037, 15.319	161	-
	UMNFO303-18	PP215001	NO: Svalbard	78.2037, 15.319	161	-
*Svalbardia lucens* (L. Koch, 1879)	UMNFO340-18	PP215023	NO: Svalbard	78.209, 15.711	11	-
	UMNFO341-18	PP215028	NO: Svalbard	78.209, 15.711	11	-
	UMNFO342-18	PP215018	NO: Svalbard	78.209, 15.711	11	-
*Fuscozetes setiger* (Trägårdh, 1910)	UMNFO412-18	PP215004	NO: Svalbard	78.040, 13.646	161	-
	UMNFO413-18	PP215019	NO: Svalbard	78.040, 13.646	161	-
	UMNFO802-19	PP215010	NO: Vestland	60.583, 7.472	1356	-
*Sphaerozetes orbicularis* (C.L. Koch, 1835)	UMNFO428-18	PP215017	NO: Vestland	60.398, 5.351	370	-
	UMNFO429-18	PP215000	NO: Vestland	60.398, 5.351	370	-
	UMNFO430-18	PP215007	NO: Vestland	60.398, 5.351	370	-
*Edwardzetes edwardsi* (Nicolet, 1855)	UMNFO462-18	PP215031	NO: Vestland	60.398, 5.351	370	-
	UMNFO463-18	PP215012	NO: Vestland	60.398, 5.351	370	-
	UMNFO534-18	PP215009	NO: Vestland	60.584, 7.519	1356	-
*Svalbardia bicuspidata* (Thor, 1930)	UMNFO490-18	PP215002	NO: Vestland	60.572, 7.478	1356	-
	UMNFO491-18	PP214997	NO: Vestland	60.572, 7.478	1356	-
	UMNFO492-18	PP215029	NO: Vestland	60.572, 7.478	1356	-
*Fuscozetes setosus* (C.L. Koch, 1839)	UZINS275-23	Na	SK	Na	Na	-
	UZINS276-23	Na	SK	Na	Na	-

**Table 2:** Measurements of some morphological characters of juvenile stages of *Fuscozetes fuscipes* (mean measurements of 4–10 specimens in μm).

Morphological Characters	Larva	Protonymph	Deutonymph	Tritonymph	Adult
Body length	343	429	540	682	512
Body width	224	273	377	475	311
Length of prodorsum	101	138	193	258	190
Length of: seta *ro*	40	47	57	62	95
seta *le*	22	24	33	49	74
seta *in*	32	64	80	93	166
seta *bs*	77	82	99	104	85
seta *c*_1_	16	24	32	41	Lost
seta *c*_2_	19	28	40	51	154
seta *c*_3_	17	29	35	44	Lost
seta *da*	14	16	28	31	Lost
seta *dp*	15	18	25	29	Lost
seta *la*	14	21	30	31	106
seta *lp*	16	23	29	29	87
seta *h*_1_	16	18	25	31	102
seta *h*_2_	37	18	23	29	89
seta *h*_3_	10	21	24	30	94
seta *p*_1_	Not developed	17	23	28	99
Genital opening	Not developed	37	49	70	114
Anal opening	87	100	121	167	163

**Table 3:** Ontogeny of leg setae (Roman letters) and solenidia (Greek letters) of *Fuscozetes fuscipes*.

Leg	Trochanter	Femur	Genu	Tibia	Tarsus
Leg I					
Larva	–	*d*, *bv″*	(*l*), σ	(*l*), *v′*, φ_1_	(*ft*), (*tc*), (*p*), (*u*), (*a*), *s*, (*pv*), (*pl*), ε, ω_1_
Protonymph	–	–	–	–	ω_2_
Deutonymph	–	(*l*)	–	φ_2_	–
Tritonymph	*v*	–	–	*v”*	(*it*)
Adult	–	*v’*	*v′*	–	*l”*, *v′*
Leg II					
Larva	–	*d*, *bv″*	(*l*), σ	*l′*, *v′*, φ	(*ft*), (*tc*), (*p*), (*u*), (*a*), *s*, (*pv*), ω_1_
Protonymph	–	–	–	–	–
Deutonymph	–	(*l*)	–	*l”*	ω_2_
Tritonymph	*v*	–	–	*v”*	(*it*)
Adult	–	*v’*	*v′*	–	–
Leg III					
Larva	–	*d*, *ev′*	*l′*, σ	*v′*, φ	(*ft*), (*tc*), (*p*), (*u*), (*a*), *s*, (*pv*)
Protonymph	–	–	–	–	–
Deutonymph	*v*	*l’* ^1^	–	*l′*	–
Tritonymph	*l*	–	–	*v’’*	(*it*)
Adult	–	–	–	–	–
Leg IV					
Protonymph	–	–	–	–	*ft′*, (*p*), (*u*), (*pv*)
Deutonymph	–	*d, ev′*	*d*	*v′*, φ	(*tc*), (*a*), *s*
Tritonymph	*v*	–	*v′*	*l′*, *v″*	–
Adult	–	–	–	–	–

Note: structures are indicated where they are first added and are present through the rest of the ontogeny; pairs of setae are in parentheses, and a dash indicates no additions. ^1^ Added in some deutonymphs; if not, this seta is added in the tritonymph.

**Table 4:** Comparison of some leg characters in *Fuscozetes fuscipes*, *F*. *coulsoni*, *F*. *kamchatkicus*, and *F*. *setiger*.

Character	*F*. *fuscipes*	*F*. *coulsoni*	*F*. *kamchatkicus*	*F*. *setiger*
Adult				
Seta *v*′ on genu I and II	Present	Present	Absent	Present
Seta *l*″ and *v*′ on tarsus I	Present	Absent	Absent	Present
Seta *l*′ on femur III	Present	Absent	Absent	Present
Anteroventral edge on femur II	Pointed	Rounded	Rounded	Rounded
Tritonymph				
Seta *v*′ on genu I and II	Absent	Absent	Absent	Present
Seta *l*′ on femur III	Present	Absent	Absent	Present

## Data Availability

The COI sequences will be publicly available in GenBank and in BOLD.
